# Wideband RCS Reduction by Single-Layer Phase Gradient Modulated Surface

**DOI:** 10.3390/s22197108

**Published:** 2022-09-20

**Authors:** Yousef Azizi, Mohammad Soleimani, Seyed-Hasan Sedighy, Ladislau Matekovits

**Affiliations:** 1 Department of Electrical Engineering, Iran University of Science & Technology, Tehran 1684613114, Iran; 2 School of Advanced Technologies, Iran University of Science & Technology, Tehran 1684613114, Iran; 3 Department of Electronics and Telecommunications, Politecnico di Torino, 10129 Turin, Italy; 4 Istituto di Elettronica e di Ingegneria dell’Informazione e delle Telecomunicazioni, National Research Council of Italy, 10129 Turin, Italy; 5 Department of Measurements and Optical Electronics, Politehnica University Timişoara, 300006 Timişoara, Romania

**Keywords:** modulated surface, phase gradient, radar cross section reduction

## Abstract

This paper deals with the design and fabrication of an unpretentious (single-layer, without any lump element) broadband (97%, 11.3–32.3 GHz) radar cross-section reduction (RCSR) modulated surface (MS). The proposed structure uses sinusoidal modulation gap sizes between square patches within square unit cells to form a phase gradient that plays an effective role in improving the RCSR bandwidth. An MS with dimensions of 250 × 250 mm^2^, consisting of 40 × 40 unit cells with a period of 6 mm printed on a RO4003C (lossy) substrate of **0.06λ_LF_** (**λ_LF_** being the wavelength at the lower frequency) thickness, has been prototyped. The MS has square patch (SP) unit cells with seven different gap sizes. A genetic algorithm (GA)-based fine-tuning has been implemented to further increase the performances of the structure. Measurements on it have been conducted considering both mono- and bi-static arrangements and for oblique incidences for both TM and TE polarization tests. A good agreement between simulation and measurement results proves the validity of the design criteria.

## 1. Introduction

A metasurface is a two-dimensional array of periodic or non-periodic structures that manipulates amplitude/phase responses at the sub-wavelength scale for wave-front steering in refraction and reflection modes [1]. These artificial sheet materials, which are usually composed of metallic patches or dielectric etchings in planar or multi-layer configurations with subwavelength thickness, have the advantages of light weight, ease of fabrication, and ability to control wave propagation both on the surface and in the surrounding free space [2]. They also enable beam shaping in both transmission and reflection. Another important application of them is to radiate in a leaky wave mode as an antenna. Other applications of metasurfaces include cloaking, polarizers, and modulators. However, the main manipulating of reflection/transmission is the major specification of metasurfaces, which make them useful in many types of telecommunications [3]. Using artificial magnetic conductor (AMC) unit cells consisting of N×N arrays (called tile) is one of the well-known methods of radar cross-section reduction (RCSR) by using a metasurface. In this method, a reflection phase difference between two or more tiles causes the RCSR. By the use of an AMC metasurface, 61% RCSR bandwidth has been reported in [4]. Moreover, bandwidth enhancement of AMC unit cells is another solution for wideband RCSR applications. To this aim, by implementation of dual- and triple-band AMCs with different resonances, 85% and 91.5% RCSR bandwidth was reported in [5,6], respectively. Using a single-layer substrate in AMC design cannot meet ultra-broadband requirements due to the low degree of freedom of design. Therefore, by the use of a multilayer substrate, an ultra-wideband AMC with improved RCSR performance is available. In [7,8], by using multilayer structures with higher complexity, RCSR bandwidth further increased up to 109% and 109.4%, in that order. Another method in the design of broadband RCSR structures is to use optimization algorithms in the design of unit cells. By pixelating the cell, the degree of freedom of design increases and wideband RCSR MS can be achieved. A pixelated checkerboard structure with 95% monostatic RCSR bandwidth was reported in [9], which has a multilayer and 0.09λ_LF_ thickness substrate. By varying the substrate thickness of tiles, which is, however, costly and difficult from a practical point of view, structures with a suitable phase gradient and RCSR bandwidth of 113%, 148%, and 122.3% were reported in [10,11,12]. Placing metasurface cells in a random configuration is known as another way to achieve broadband RCSR performance. For this purpose, the use of a metasurface in a random configuration resulted in 73% and 77% RCSR bandwidth in [13,14], in turn. Using holographic surfaces to convert the impinging wave into surface one, as well as anomalous or diffuse reflection of the incidence, narrowband RCSR can be achieved, as reported, for example, in [15,16,17]. The phase gradient metasurface (PGM) is a special appearance of MS, which has been proposed by Yu et al. to demonstrate the generalized Snell’s law [18]. PGM have been highlighted as a good candidate for realizing electromagnetic-wave-focusing characteristics by manipulating the wavefront through controlling the spatial phase and transmission profiles of metasurfaces. Since the PGMs are able to provide pre-defined in-plane wave vectors to manipulate the directions of the refracting/reflecting waves, it consequently attracts a lot of attention in such a kind of beamforming mechanism [19,20,21] and RCSR [22,23,24].

For improvement of RCSR bandwidth, PGM structures with anomalous reflection in [22,23] reports 61.2% and 82.4% RCSR bandwidth, respectively. Using a large number of unit cells with amplitude and phase gradient in a sinusoidal modulated (SM) configuration led to 128% RCSR bandwidth with a substrate thickness of 0.1λ_LF_ [24]. The 6 dB RCSR bandwidth, which covers frequencies in the 5–34 GHz range, is proposed in [25], which uses three-unit cells with proper phase difference between them. Even though the MS has a suitable 5 dB RCSR bandwidth, the 10 dB RCSR bandwidth of structure is limited to lower 50%, which is considered as a structural drawback. According to the literature review stated above, there is always a trade-off between the RCSR bandwidth and the simplicity and cheapness of the MS structures. In order to achieve a wideband RCSR MS, the cost and difficulty of its design process should also be taken into account. The design of a single-layer, simple, low-cost metasurface with large RCSR bandwidth is always a challenge for designers and researchers. As a conclusion from the discussion, it can be said that the design of a simple, single-layer, low-cost and also wideband RCSR MS is always a challenge for researchers, and in this paper, the authors aim to provide a solution in this regard.

In this paper, a square patch (SP) unit cell with non-uniform gradient reflection phase by changing the gap size is perceptively placed in the structure giving rise to sinusoidal modulation so that wideband RCSR bandwidth is achieved. This is because using a non-identical number of unit cells is easily possible with sinusoidal modulation. In this phase of the design, genetic algorithm (GA)-based optimization has been considered. The use of GA is an effective solution in the implement of SP unit cells with phase gradients in a single-layer MS, which is able to scatter the normal incident wave in other directions; consequently, wideband RCSR is achieved. Finally, the measurement results indicate 97% of the monostatic 10-dB RCSR bandwidth for normal incidence and 54% and 45% for TM and TE oblique incidences (15°–40°). Low profile (0.06λ_LF_ substrate thickness—**λ_LF_** being the wavelength at the lower frequency), simple structures, and wideband RCSR performance are the advantages of the proposed structure compared with state-of-art references.

The paper is structured as follows: design concept and implementation procedure are presented in Section 2. Identification of the best configuration, that has been prototyped, and comparison between measurements and numerical data with deep discussion about the results are presented in the Section 3. The final section is devoted to the conclusions.

## 2. Design Concept

The RCSR value in AMC structures that have two single cells is calculated by the following Equation [4]:

(1) $$RCSR\;\left(dB\right)\;=\;10\;log\;{\left|\frac{\left|\Gamma_{1}\right|e^{j∡\Gamma_{1}}+\left|\Gamma_{2}\right|e^{j∡\Gamma_{2}}}{2}\right|}^{2}\;.$$

where, |$\Gamma_{1}$|, |$\Gamma_{2}$|, $∡\Gamma_{1}$, and $\;∡\Gamma_{2}\;$ are the reflection amplitude and phase from the unit cells of type 1 and 2, respectively.

According to Equation (1), if the reflection amplitude of both unit cells is equal to 1, the phase difference of 180° ± 37° between them causes an RCSR of at least 10 dB [24]. It is relatively challenging to design two simple, single-layer unit cells that have the desired phase difference in wideband [16,24], and therefore, more unit cells should be used according to [22,23,24,25], to have a smaller phase difference with respect to each other. If *n* types of cells are used, where each of them has its own number of repetition (*m_i_*) and they totally reflect the incident waves, i.e., |*Γ_i_*| = 1, (*i* = 1, …, *n*), Equation (1) is expressed as [24]:

(2) $$RCSR\;\left(dB\right)\;=\;10\;log\;{\left|\frac{m_{1}e^{j∡\Gamma_{1}}+\;m_{2}e^{j∡\Gamma_{2}}+\;.\;.\;.\;+\;m_{n}\;e^{j∡\Gamma_{n}}}{\sum_{1}^{n}m_{i}}\right|}^{2}.$$

**Figure 1:**
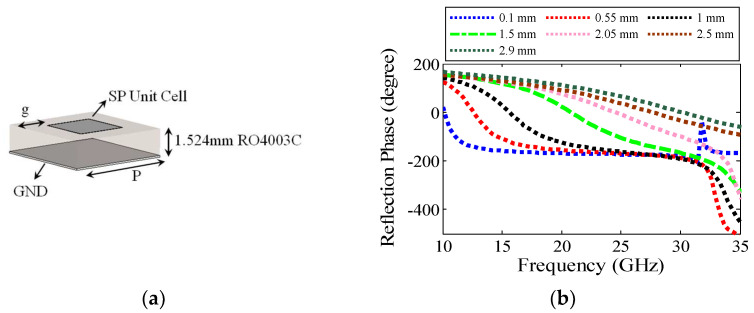
SP unit cell and reflection phase (**a**) 3D schematic of SP unit cell; (**b**) reflection phase of the SP unit cells with period equal to 6 mm for different gap width g.

In the above Equation (2), the reflection phase and the number of unit cells from each type (*m_i_*) are the effective variables in RCSR. In fact, the number of each cell as a weighting factor can help increase the RCSR bandwidth. The 3D schematic view and reflection phase of SP unit cells for gap size variation (*g*) from 0.1 mm to 2.9 mm is plotted in Figure 1a,b, respectively.

The SP unit cells were designed on a grounded RO4003C substrate (ε_r_ = 3.55) with 0.06λ_LF_ thickness and a period of 0.22λ_LF_. The used substrate has relatively low losses (loss tangent of 0.0027) and its dispersion effects can be ignored, so the reflection amplitude of the SP cells in the frequency range of 10–35 GHz is approximately equal to 1 (|*Γ_i_*| = 1). It should be noted that CST Suite has been used for full wave analyses of SP unit cells of Figure 1. In more details, periodic boundary condition has been chosen for the unit cell; moreover, in the normal to the unit cell surface direction open space boundary condition at 10 mm has been considered. A CST frequency solver with mesh refinement (for high accuracy results) properties has been used for full wave analysis of unit cells.

**Figure 2:**
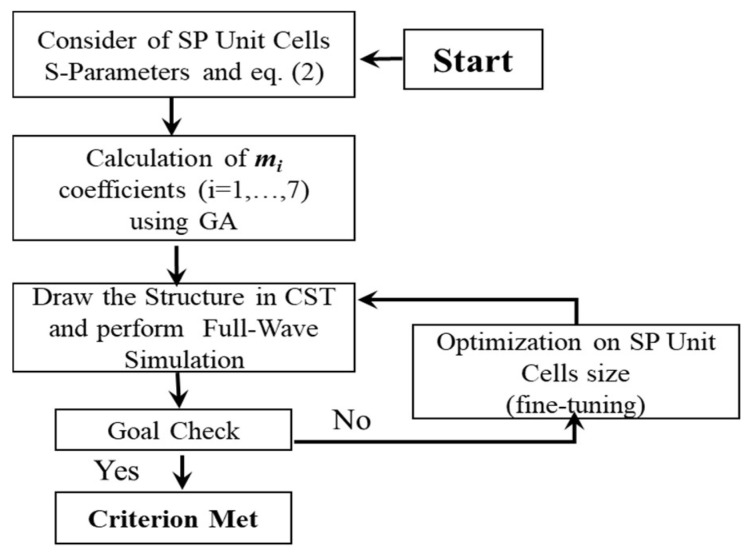
Block diagram of MS design and *m_i_* calculation with GA.

It is observed that the reflection phase difference between adjacent cells (by decreasing *g*) is not uniform in the whole 10–35 GHz band. For a small-sized gap (0.1–1 mm), a relatively suitable phase difference between the SP cells is formed at low frequencies (10–20 GHz), where the reflection phase of these cells is similar to the high frequency answer. On the other hand, with an increasing gap (1.5–2.9 mm), the desired phase difference between SP cells is formed at higher frequencies (20–35 GHz), and the phase difference at lower frequencies is not satisfying. However, in some references, e.g., [11,22,26], by using a coding metasurface, substrate thickness change, as well as unit cell rotation, wideband RCSR structures with relatively uniform phase differences between unit cells have been reported; however, they are costly and complex. In fact, the existence of the equal phase difference between adjacent cells has a more effective role in maximum RCSR bandwidth enhancement. A block diagram for implementation of SP unit cells with non-equal weight coefficients (*m_i_* in Equation (2)) arranged in the MS structure is shown in Figure 2. Based on Figure 2, in the first step, the SP unit cell’s S-parameters (reflection phase) of Figure 1 are considered as inputs of GA. In the next step, the repetition number (*m_i_*, *i* = 1, …, 7) of each cell is considered as an unknown variable for GA. In the third step, the MS structure is drawn in CST and full wave analysis is done. Since the GA procedure tried to minimize an Err. function as a goal check requirement, the $\frac{1}{BW}$ considered as an Err. function was checked every time. After this goal of checking whether the BW enhancement is not satisfied, then the unit cell’s size and *m_i_* numbers should be fine-tuned and MS structure redrawn in CST. By implementing these steps hundreds of times, then the suitable coefficient number of SP unit cells (*m_i_*, *i* = 1, …, 7), which corresponds with the maximum 10 dB, the RCSR bandwidth is extracted.

**Figure 3:**
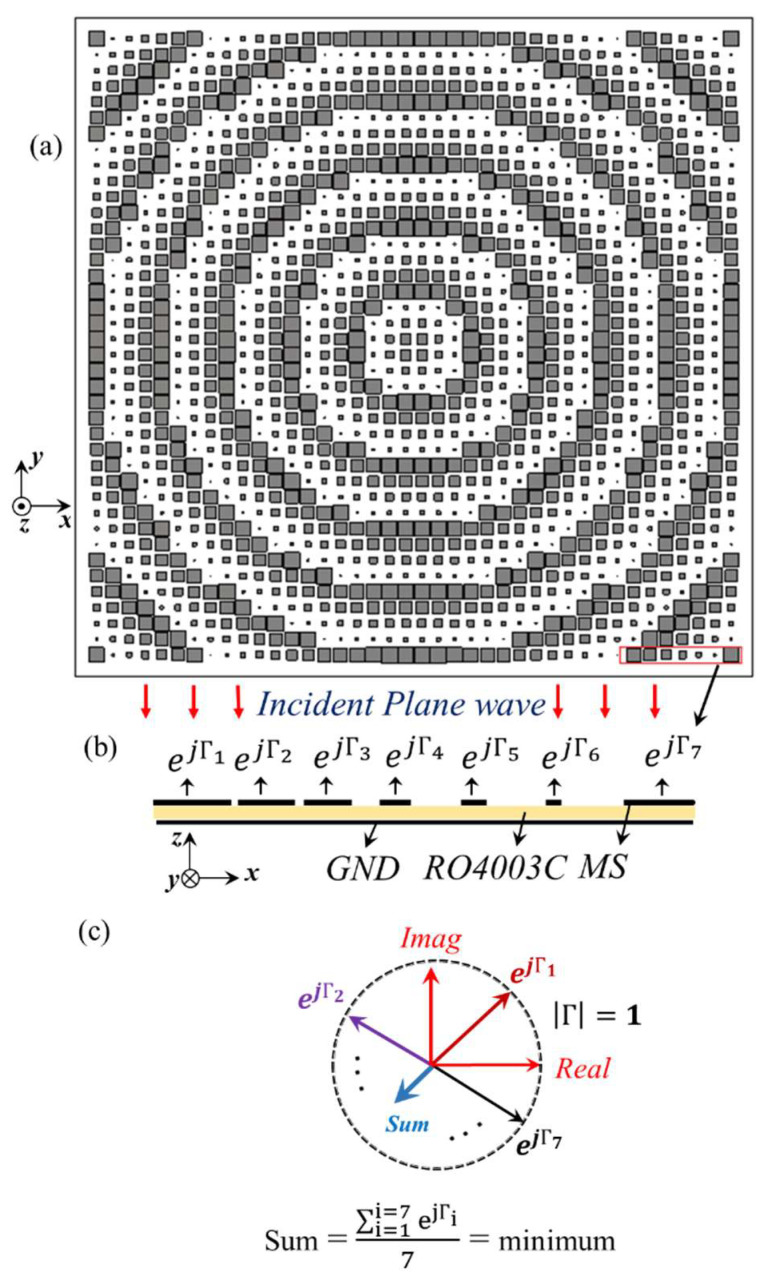
Schematic of PGM structure and RCSR mechanism: (**a**) PGM structure; (**b**) PGM surface performance when illuminated by incidence plan wave; (**c**) RCSR mechanism of PGM by phase cancellation with reflections of different unit cells which shown with colorful arrows.

By implementing the GA, we are able to target the fine tuning of the arrangement of SP cells in the MS configuration in Matlab and CST Microwave Studio (Figure 2). The design formula for the implementation of SP unit cells in the SM arrangement is as below [24]:

(3) $$g_{m,n}=A\times \left|\sin\left(w\times r_{m,n}+\varphi \;\right)\right|$$

where, *A*, *w*, *φ* are auxiliary variables and $r_{m,n}=\sqrt{m^{2}+n^{2}\;},$ ( m,n=1, …, 13) is the distance of the (*m, n*)th cell from the center of MS, expressed in the 2D Cartesian (x/y) coordinate system. Furthermore, *g_m,n_* is the gap size of the (*m*, *n*)th cell. The GA was implemented to minimize the sum reported in Figure 3c, and the values of A=2.9, w=31 ,$\;\varphi =-40^{o}\;$ have been obtained, which have been used to generate the PGM structure in Figure 3a. Due to the symmetry with respect to the X/Y coordinates, the sinusoidal modulation has a symmetrical RCS pattern (with respect to x/y axis) that prevents the formation of the main lobe in an undesired direction, so it is suitable for RCSR application. Figure 3a shows a schematic of the wideband MS that consists of SP unit cells (with the dimensions of Figure 1). Figure 3b shows the reflection performance of a part of the structure when illuminated by an incident plan wave. Using low loss **0.06λ_LF_**, a thin substrate causes the reflection of the entire incidence wave. Therefore, the reflection phase is the main parameter for wideband RCSR achievement (Figure 3c). SP unit cells with a gap size (*g*) of 0.1, 0.55, 1, 1.55, 2.05, 2.5, and 2.9 mm were placed in the MS configuration of Figure 3a with the *m_i_* (*i* = 1, …, 7) coefficient of 104, 112, 164, 196, 412, 336, and 276, respectively. These coefficients are the optimal values obtained by GA that was applied after having the cost function in Equation (2) satisfied. In the GA procedure, the reflection phase of SP unit cells (Figure 1) is considered as inputs and *m_i_* (coefficient of each SP cell) is considered as an unknown parameter that potentially leads the Err function to be minimized.

## 3. Discussion and Results

**Figure 4:**
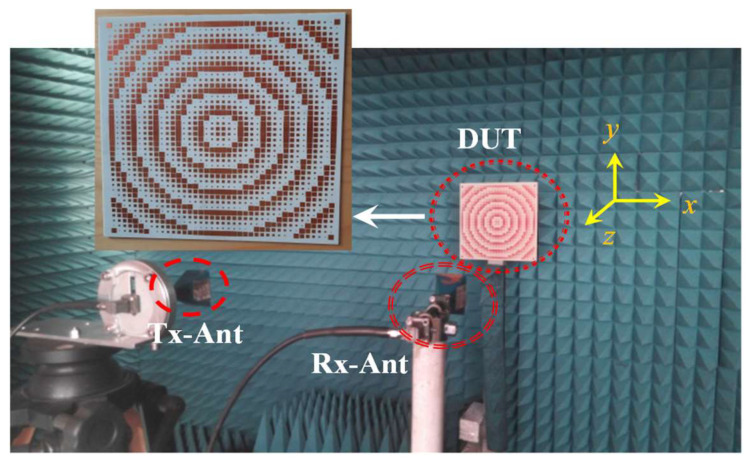
Fabricated structure (DUT) and RCS test set-up (Tx/Rx antenna in anechoic chamber).

Figure 4 shows the prototype of the proposed broadband RCSR MS that is printed on a RO4003C (lossy) substrate with dimension of 250 × 250 mm^2^ and composed of 1600 unit cells.

RCS measurements have been carried out in an anechoic chamber using an N5227A PNA network analyzer as a source and receiver. The 30 GHz test bandwidth (10–40 GHz) is covered by three sets of Tx/Rx antenna. The test method is based on time gating and it needs post-processing of the initial results to extract the final values (see [27]). Two antennas are used to transmit and receive the chirp signals of the PNA, as reported in Figure 4. The distance between antenna and structure is 4 m. In the measurement procedure, the Tx antenna is placed in the front of the PCB/GND of the proposed MS, and the Rx antenna is moved manually at the same time on the rotating angular calibrated arm to extract the reflection information at all angles from 1° to 90° for different frequencies. Then, the PCB/GND measurement information at each angle is post-processed, by converting them from frequency to time domain by using inverse fast Fourier transform (IFFT). The data are then time filtered and re-converted to the frequency domain by using FFT. The procedure is applied for both PCB and GND reflection results at each angle and the bi-static pattern is consequently extracted at each frequency. Since the measurement methods and numerical tools of CST Suite software are in good agreement with the IEEE 1502 recommended [28] (known as the main reference for RCS measurement), the measurement results of the proposed method are compared and validated with the simulation ones.

**Figure 5:**
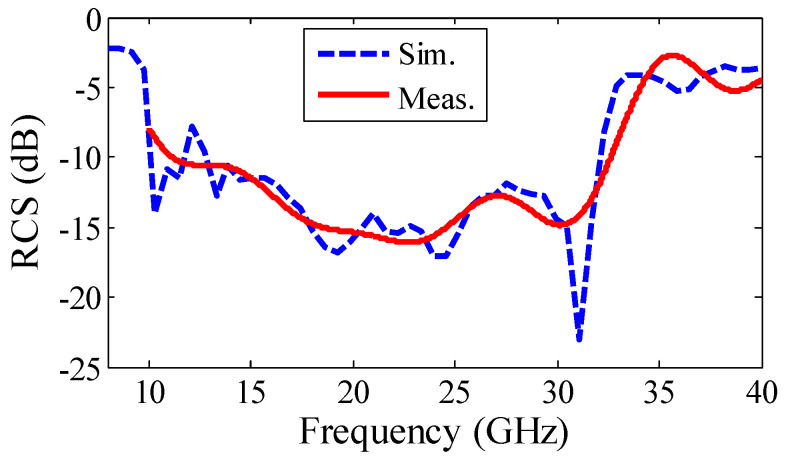
Monostatic RCS results of the PGM structure.

**Figure 6:**
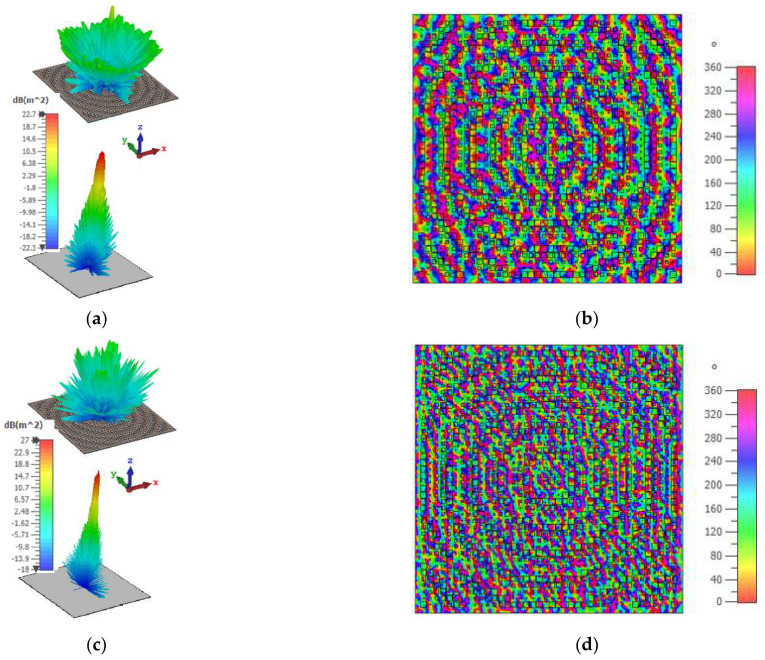
RCS pattern of PGM with the PEC of the same size and 2D E-Field phase distribution of the structure: (**a**) 18 GHz RCS pattern; (**b**) 18 GHz phase distribution; (**c**) 31.1 GHz RCS pattern; and (**d**) 31.1 GHz phase distribution.

The measured result of the monostatic RCSR structure shown in Figure 5 verifies the simulation one. Based on the measured results in the frequency range of 11.3–32.3 GHz (97%), an RCSR of more than 10 dB is achieved. Wideband 10–40 GHz RCS results of the MS were extracted from three measurements (in three separate frequency bands of 10–18 GHz, 18–22 GHz, and 22–40 GHz), and these are merged in one single plot, which is depicted in Figure 5. For an RCS measurement of each frequency band, the number of frequency points and the PNA resolution bandwidth is chosen as 1000 and 100 Hz, respectively. By using these settings and also by using the Kaiser filter, the extraction of RCS results is possible. A comparison between the simulation and measurement results of Figure 5 shows that by using SM and applying intelligent weighting coefficients in a SP unit cell, wideband RCSR performance can be achieved. The RCS pattern and also 2D phase distribution of the structure at 18 GHz and 31.1 GHz, are shown in Figure 6. Results in Figure 6 demonstrate that a large part of the incident plane wave is redirected in different directions by scattering the input wave. The oscillations between the measurement and simulated results are due to tolerances in fabrication, and accuracy of the post-processing, i.e., extracting the results from the time gating method and applying time filters. The highest difference can be noted around 31.5 GHz. However, the general behavior is followed in a wide frequency range.

**Figure 7:**
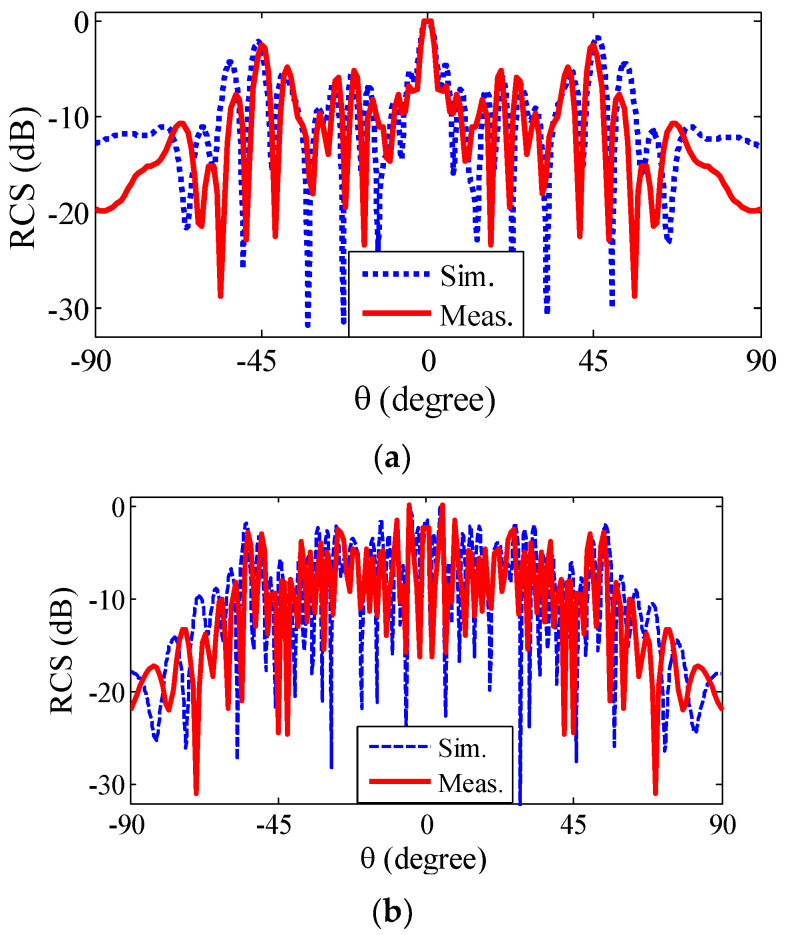
Bi-static RCS results: (**a**) 18 GHz; (**b**) 31.1 GHz.

As shown in Figure 6a,c, at 18 GHz and 31.1 GHz the structures (compared with the PEC of same size) redirects the reflection: most of the redirection main lobes occur at 45° and 24° angles, respectively, which can be observed in the Cartesian plots in Figure 7. Figure 6a,c demonstrates that the proposed MS effectively scattered an incident wave in the other direction. Moreover, the proposed MS, which was designed by GA, has a wideband −10 dB RCS compared with a PEC of the same size. Furthermore, Figure 6b,d demonstrates that the MS has SP cells with various reflection phases (between 0°–360°), which play an effective role in phase cancellation and RCSR achievement. Figure 7a,b shows a 2D RCS measurement and simulation pattern of the structure at 18 and 31.1 GHz, respectively. The measurement of the bi-static RCS patterns is done with an angular step of 1° (due to equipment limitation), and therefore, there are some rapid changes in the measurement; the simulation results have an accuracy of 0.25°. As shown in Figure 7a, the 2D RCS pattern is perfectly symmetrical, and at 18 GHz at angles of 1°–60°, there is good agreement between the simulation and measurement results, but from 70° to 90° there are some inconsistencies that can be caused by manufacturing errors. The reason for this difference can be due to environmental factors, such as set-up vibration, openness in the chamber gate, and errors related to the construction of metasurfaces and the calibration of test equipment. In general, the good similarity that exists between the simulation and measurement results in 18 GHz is due to the following reasons: (i) an accurate angular calibration that allowed for the accurate measurement of reflection at any angle; (ii) the elimination of the reflection noise effect from the environment by the time filter, which reduces a significant part of the environmental measurement error (an intrinsic advantage of time gating method). Similarly, at 31.1 GHz, although the oscillation rate in the pattern increases with increasing frequency, there is a good agreement between the measurement and simulation results at all angles from 1° to 90° (Figure 7b). Unlike the simulation results at the 18 GHz frequency and 60°–90° incident waves angles, there is a good agreement between the measurement and simulation results.

**Figure 8:**
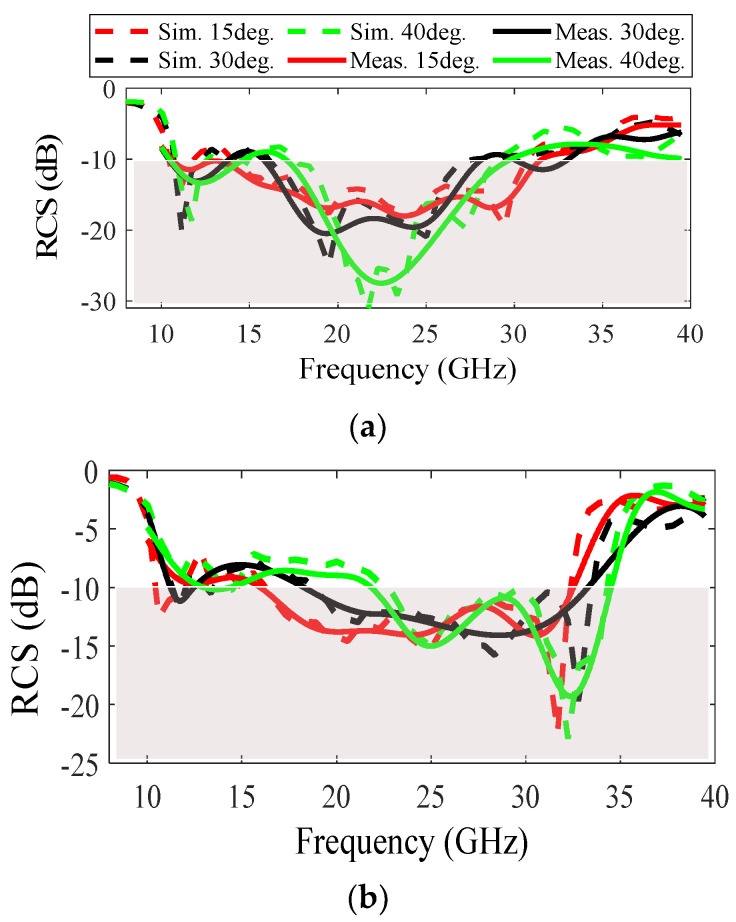
Sensitivity study of the proposed PGM for oblique incidence: (**a**) TM polarization; (**b**) TE polarization.

The proposed PGM MS has a low sensitivity to oblique incidence (15°–40°), as shown in Figure 8a,b, for TM and TE polarizations, respectively. Since the reflection phase of the SP unit cell for oblique incidence is more sensitive to TE polarization (compared with the TM one), it is expected that its RCSR bandwidth will be more sensitive to TE polarization compared with the TM mode [29]. By increasing the incidence angle from 15° to 40°, RCSR bandwidths of 45% (21.7–34.5 GHz) and 54% (17.5–30 GHz) are consequently achieved for TE and TM polarizations, respectively. The comparison between the proposed wideband RCSR MS and the state-of-the-art references is presented in Table 1. Although [7,8,10,11,12,24,30] have RCSR bandwidth larger than the proposed structure, they are complex and costly to build due to the use of substrates with different thicknesses; additionally, they are multilayer structures. The use of multilayer substrates increases the cost and difficulty of the assembly process. Simplicity of design (single layer), no need for spacers and lump elements, wideband RCSR (97%), and low substrate thickness (0.06λ_LF_) are the advantages of the proposed structure. The RCSR bandwidth of 97% of the proposed structure is highlighted, compared to that referenced in Table 1, while the proposed structure has a lower substrate thickness (0.06λ_LF_) compared to others, which is considered an advantage.

The presented results of the proposed MS prove that the use of GA with sinusoidal modulation could be helpful in designing new novel RCSR structures with novelties compared to the state-of-the-art references.

In the GA procedure, the right description of inputs (in this case SP unit cells with different dimension, reflection phase, and repletion factor) and the relation between inputs/output (in this case RCS difference between PEC and MS) are the two critical points. By considering these facts, this paper presents a simple MS, which uses a sinusoidal modulation as an approach for wideband-effective phase cancellation achievement between SP cells, that results in 97% of monostatic RCSR. It could be expected that a redesign of the proposed GA procedure with the use of other types of modulation between unit cells may be helpful in designing new novel structures in RCSR, antenna, and other fields of telecommunication.

## 4. Conclusions

In this paper, a wideband (97%) RCSR MS is presented, which is of reduced complexity, single-layer, and has low sensitivity to inclined illumination for both TM and TE polarizations. In the proposed sinusoidal modulated structure, each SP cell with its own repetition number obtained by GA was used to achieve effectively wideband RCSR with a symmetry of pattern. The placement of SP cells with different dimensions in the sinusoidal modulation configuration makes the bandwidth of RCSR increase effectively. A 2D phase distribution of the MS in two sample frequencies demonstrates that the arrangement of SP cells in the MS configuration leads to the formation of a 0°–360° phase difference, which is critical for RCSR bandwidth enhancement. Furthermore, an example of the fabricated structure is measured in cases of mono- and bi-static answers, for oblique incidence for both TM and TE polarizations. The RCSR bandwidth of the proposed MS, with an oblique incidence angle of 15°–40° for both TE and TM polarization, is equal to 45% and 54% (of fractional BW), respectively. The experimental measurement of RCSR has been performed using the time gating method, which increases the accuracy of the measurement by removing the noise and effect of the field received by the side lobe of the transmitter/receiver antennas. The presented experimental results are in good agreement with the simulation results, which verifies the design procedure.

## Figures and Tables

**Table 1:** Comparison between the proposed PGM and the state-of-the-art references.

Structure	Thickness (λ_LF_)	10 dB BW (%) FrEquation (GHz)	No. of Layers	Substrates
[6]	0.14	91.5/3.77–10.14	2	RO4350B-Air
[7]	0.1	109/13.1–44.5	3	FR4/air
[8]	0.1	109/4.8–16.4	2	FR2-Air
[10]	0.13	113/6.4–44.5	2 uneven layers	F4B
[11]	0.125	148/6.16–41.3	3 uneven layers	F4B
[12]	0.124	122/6.2–25.7	3 uneven layers	F4B
[23]	0.071	82.4/7–16.8	1	RO5880
[24]	0.1	128/9–40.7	2	FR4
[26]	0.075	91/15–40	1	F4B
[30]	0.11	108/13.6–45.5	2	FR4-FR4
This work	0.06	97/11.3–32.3	1	RO4003C

## Data Availability

Not applicable.
